# Understanding the Second Victim Phenomenon Among Healthcare Workers in an Italian Hospital

**DOI:** 10.3390/ejihpe14120201

**Published:** 2024-12-16

**Authors:** Raffaella Sedile, Antonella Zizza, Luca Bastiani, Eugenia Carluccio, Marinella Marrazzi, Tommaso Bellandi, Giorgio O. Spagnolo

**Affiliations:** 1 Institute of Clinical Physiology, National Research Council, 73100 Lecce, Italy; raffaella.sedile@cnr.it (R.S.); antonella.zizza@cnr.it (A.Z.); 2 Institute of Clinical Physiology, National Research Council, 56100 Pisa, Italy; 3 Medical Management, Vito Fazzi Hospital Lecce, 73100 Lecce, Italy; 4 Emergency Department, Vito Fazzi Hospital Lecce, 73100 Lecce, Italy; 5 Patient Safety Unit, Northwest Trust, Regional Health Service of Tuscany, 50139 Firenze, Italy; 6 Institute of Information Science and Technologies, National Research Council, 56100 Pisa, Italy; giorgio.oronzo.spagnolo@isti.cnr.it

**Keywords:** second victim, medical errors, adverse event, second victim syndrome, second victim phenomenon, SVEST, coping

## Abstract

Second victim syndrome (SVS) refers to the psychological trauma experienced by healthcare workers (HCWs) as a result of being involved in an adverse event (AE). Research on the prevalence of SVS and the support needed for HCWs who experience it is limited. A cross-sectional study was conducted at the Health Local Unit of Lecce, in Puglia, to identify the phenomenon of SVS among HCWs and recognize the forms of support received and desired. A validated questionnaire, IT-SVEST, was administered to doctors and nurses. The survey received responses from 250 HCWs, and 41% of respondents reported being involved in an AE that could cause SVS. Among the seven dimensions measuring the effects of the SVS and two outcome variables, the highest percentage of agreement was found for psychological distress (23.5%), followed by turnover intentions (19.8%) and physical distress (9.9%); 23.8% of the interviewees declared that they did not receive institutional support, and 9.9% identified help resources mostly in non-work-related support (9.9%), followed by supervisor support (9.3%). The multivariable binary logistic regression analysis showed a positive association between the occurrence of an AE and the medical doctor profession (OR = 4.267, *p* ≤ 0.0001), and affiliation to intensive care departments (OR = 5.133, *p* ≤ 0.0001) and male gender (OR = 2.069, *p* = 0.033). SVS is a serious problem that affects the entire health system, systematic surveys and appropriate institutional responses including formal support programs for affected HCWs are a priority.

## 1. Introduction

Second victim syndrome (SVS) is a latent phenomenon within healthcare organizations, with limited evidence in terms of prevalence, etiology, and effects, but with a strong qualitative and economic impact on the healthcare system. The term “second victim” was coined by Wu in 2000 to describe the experience of healthcare workers (HCWs) directly or indirectly involved in an unexpected and unintentional error that has negative consequences for both the patient and HCW, who remains traumatized by the event itself [1].

SVS represents the psychological trauma that HCWs experience due to involvement in an adverse event (AE) in an era when modern medicine generates expectations for a “zero error” standard for doctors [2]. After an AE, the HCWs may experience different outcomes: thriving, surviving, or dropping out. Some HCWs may exhibit constructive coping behaviors, such as improved communication skills and greater awareness, resulting in an overall improvement in their practice. Other HCWs may show maladaptive coping behaviors characterized by feelings of guilt and anxiety, such as the hypervigilance that produces excessive attention to detail, constant alertness, and fear of making further mistakes. Still, others, finding it difficult to continue caring for patients, may choose to abandon the profession [3,4,5].

Many studies have shown that the most frequent emotional responses to AE include anxiety, loss of self-esteem, feelings of guilt, worry, nervousness, anger, frustration, and lower professional confidence [6,7,8,9]. Additionally, major concerns are fear for the patient, the possibility of disciplinary action, and potential punishment [8]. Psychological repercussions, such as dissatisfaction, burnout, emotional exhaustion, depersonalization, and symptoms of depression [10], are often accompanied by physical symptoms such as sleep disorders, frequent nausea, and increased blood pressure and respiratory rate [11,12].

Recent articles have highlighted that the syndrome can result not only from actual medical errors but from any incident related to patient safety, regardless of the severity of its consequences [13,14].

More than half of HCWs have experienced SVS at least once in their lives and reported needing support [15,16,17]. Most preferred to discuss with a colleague or peer, or with a family member, friend, or supervisor [15].

Previous studies have indicated that self-disclosure to colleagues can effectively reduce emotional distress after a medical error [4,18], and peer debriefings are considered valuable support tools since they provide reassurance, validate competence, and help develop resilience strategies [19].

To date, limited research is available to evaluate the phenomenon of SVS and the type of support needed for HCWs who suffer from it. In a recent study among respiratory therapists at a North American healthcare organization, 91.2% of the study participants reported having been involved in a work-related stressful or traumatic event, and 57.7% of these identified as second victims, experiencing anxiety in 39.1% of subjects, insomnia in 32.1%, and guilt in 28.2% [20].

The Department of Obstetrics and Gynecology is one of the clinical specialties most prone to stressful and traumatic events [21], probably because the EA can involve more than one victim: a mother and a child. A survey conducted among professionals in maternity care revealed that 47.8% of participants had experienced the second victim phenomenon during their careers. The main causes included patient accusations (84.6%), unexpected complications (59.0%), colleague complaints (56.4%), disputes (43.6%), missed diagnoses (33.3%), and patient deaths (33.3%). Nearly all healthcare workers (95.5%) reported receiving support from their colleagues [22].

In an Asian study, 93.3% of HCWs who experienced an AE with significant emotional impact (sadness, guilt, and anxiety) reported not receiving counseling after the event; 72% of them did not consider it necessary to receive any support [23].

Many HCWs do not seek counseling after AE, due to difficulty finding time, loss of confidentiality in the event of legal action, and fear of being negatively judged by colleagues [24].

A recent systematic review showed that most hospitals and medical centers worldwide lack organized support interventions, although there is increasing recognition of the need to build second-victim response teams [25]. The consequences of the SVS are not limited to the health and the actions of the HCWs involved but can trigger a chain reaction that affects the entire healthcare organization, resulting in malfunction, negative reputation, and waste of economic resources [26].

Burlison et al. developed and validated a brief quantitative screener, the Second Victim Experience and Support Tool (SVEST), to standardize key definitions and estimate the Second Victim Experiences and Support resources [27]. This tool has been recently adapted and validated in several countries [28,29,30], including Italy [31,32].

Our study aims to explore the lived experiences and the support received by HCWs involved in an AE. To this end, we assessed the sociodemographic characteristics of the interviewees to identify any factors related to the event, and we examined the psychological, physical, and behavioral effects to better characterize symptoms associated with the second victim experience. Furthermore, we investigated the forms of support received and desired to inform and promote an appropriate and supportive institutional response.

## 2. Materials and Methods

The study was conducted at Vito Fazzi Hospital in Lecce, Italy, the largest hospital in the province, serving a population of 771,230 inhabitants [33].

Our study collected information through convenience sampling. All hospital departments were informed about the study through an institutional email sent by the health management. Medical and nursing staff employed at the hospital for more than one year were invited to participate in the anonymous survey. Participants received a brief description of the survey, an explanation of the potential risks and benefits, and a paper questionnaire to be completed. The completed questionnaire was returned in a sealed envelope without any identification information. Informed consent was obtained from all HCWs who agreed to take part in the research study.

The nursing coordinators of the various departments were responsible for handling the reminder and collection activity. The completed questionnaires were collected from each department, and the answers were entered into a database. The Ethics Committee of the ASL Lecce approved the study with Prot. N. 0000484 of 2 December 2022.

The survey received responses from 250 HCWs out of 708 invited to participate, belonging to 16 of the 45 departments of the hospital.

The study utilized IT-SVEST, a questionnaire validated in Italian for healthcare personnel, including doctors and nurses. The self-administered questionnaire was divided into four sections. The Section 1 consisted of four questions to evaluate the occurrence of a second victim event (SVE). The Section 2 included 29 items of the SVEST, organized into 7 dimensions (psychological distress, physical distress, support for colleagues, supervisor, organization, non-work-related support, and professional self-efficacy) and 2 outcome variables (intentions to turnover and absenteeism). The Section 3 included six questions to investigate the preferred forms of support. Respondents were asked to express their level of agreement with the 29 items about their personal experiences using a 5-point Likert scale ranging from 1 = strongly disagree to 5 = strongly agree. The responses to SVEST were analyzed according to published instructions [25], with the degree of agreement per item as the average of responses equal to or greater than four on the scale. Mean scores were calculated only for respondents who answered more than 50% of the specific items on that variable (e.g., ≥3 of 4 items, ≥2 of 3 items, or both of 2 items). A higher score in each dimension indicates greater levels of psychological distress, physical distress, lower professional self-efficacy, and a greater perception of support received. Higher scores for each outcome indicate higher turnover intentions and absenteeism. The Section 4 and Section 5 included five questions that examine sociodemographic characteristics with a range of answer options.

### Statistical Analysis

The statistical analyses were conducted using SPSS Version 24 (IBM Corp, Armonk, NY, USA) or Stata/SE 13.1, with a significance level set at *p* < 0.05. Categorical variables were expressed as percentages, while continuous variables were reported as mean ± standard deviation (SD). Descriptive analyses were performed to summarize the sociodemographic characteristics of HCWs enrolled in the study. For comparison between categorical and nominal variables, the Pearson chi-square and Fisher’s exact test were used, while for the evaluation of continuous variables, the Student’s t test was employed. Multivariate logistic regression analyses were performed to determine the independent association of potential predictor characteristics with the SVE.

Multivariate logistic regression is the most suitable statistical modeling technique for predicting the probability of a binary event (e.g., yes/no, true/false) based on a set of independent variables. In this context, our binary event (dependent variable) was classified as 0 for subjects who did not report an adverse event and as 1 for those who did. The independent variables included in the model consisted of common information from both groups—those with and without the event. These variables included gender, age group, profession, work area, and years of experience.

The analyses included all significant variables identified in the univariate analyses. The final model was selected using a stepwise backward analysis, and the results were presented as an adjusted odds ratio (OR) with a 95% confidence interval.

Additionally, among the participants who reported the adverse event, a confirmatory factor analysis (CFA) was performed using Structural Equation Modeling (SEM) in Stata/SE 13.1. This analysis enabled us to simultaneously examine the interrelationships among various variables. The model explored and described the causal relationships between four latent variables: SVEST, absenteeism, turnover, and socio-demographic characteristics.

Overall model fit was assessed using the Root Mean Square Error of Approximation (RMSEA), where values between 0.05 and 0.08 indicate acceptable fit and values < 0.05 indicate good fit; the Comparative Fit Index (CFI) and Tucker-Lewis Index (TLI) with values > 0.90 indicate reasonable fit and > 0.95 indicate good fit; and the Standardized Root Mean Square Residual (SRMR) with values < 0.10 indicating good fit [34].

## 3. Results

Out of the 708 HCWs invited to participate in the survey, 250 responded, yielding a response rate of 35%. Of these, four questionnaires were excluded due to missing data.

Among the interviewees, 71.1% were female, 54.7% were operators who worked in non-surgical and non-intensive care departments (medical area), and the majority were nurses (80.7%), aged between 31 and 50 (51.4%) and with over 15 years of professional experience (45.0%).

Furthermore, 101 respondents (41.0%) reported being involved in an AE related to clinical or healthcare deficiencies that had consequences for one or more patients. Only one of these incidents was classified as a “near miss”.

HCWs involved in an AE were mostly women (60.6%), nurses (70.0%), and from the medical area (51.5%) (Table 1).

The agreement percentages, means ± SD for seven dimensions, and two outcomes of the I-SVEST, based on the score instructions of Burlison et al. [27], are presented in Table 2.

Among the dimensions measuring the effects of the SVS (with higher scores indicating greater effects), the highest percentage of agreement was found for “Psychological Distress” (23.5%), followed by “Turnover Intentions” (19.8%) and “Physical Distress” (9.9%).

Regarding the categories of the IT-SVEST that analyze the forms of support received, 23.8% did not receive “Institutional Support”, while 9.9% identified help resources mostly in “Non-Work-Related Support” (9.9%), followed by Supervisor Support” (9.3%).

In the survey, respondents indicated the main effect of SVS was psychological distress. 55.5% of the participants felt “miserable” after the AE (Question 3), and 54.0% felt embarrassed about the accident (Question 1). Furthermore, 51.5% of participants reported that “the mental burden of my experience is exhausting” (Question 5). Some of them also experienced symptoms of physical distress, such as difficulty sleeping regularly (33.7%—Question 6), lack of appetite (14.9%—Question 8), and feeling queasy or nauseous (23.8%—Question 7).

Regarding the support received, the highest scores were obtained for the statements “Discussing what happened with my colleagues provides me with a sense of relief” (Question 10; 78.2%) and “My colleagues help me feel that I am still a good healthcare provider despite any mistakes I have made” (Question 12; 75.0%). The sentence “The love from my closest friends and family helps me get over these occurrences” (Question 21) received a positive response from 70.3% of interviewees.

However, the survey results indicate a lack of support and interest from the institution regarding the second victim phenomenon. Only 22.8% of the interviewees believe that “My organization offers a variety of resources to help me get over the effects of involvement with these instances” (Question 18); 54.5% stated that “The concept of concern for the well-being of those involved in these situations is not strong at my organization” (Question 19).

Only 21.8% stated that “My experience with these events has led to a desire to take a position outside of patient care” (Question 26) (Table 3).

Finally, 78.2% of respondents stated that the preferred form of support (Question 4) was “An employee assistance program that can provide free counseling to employees outside of work” (Table 4).

**Figure 1:**
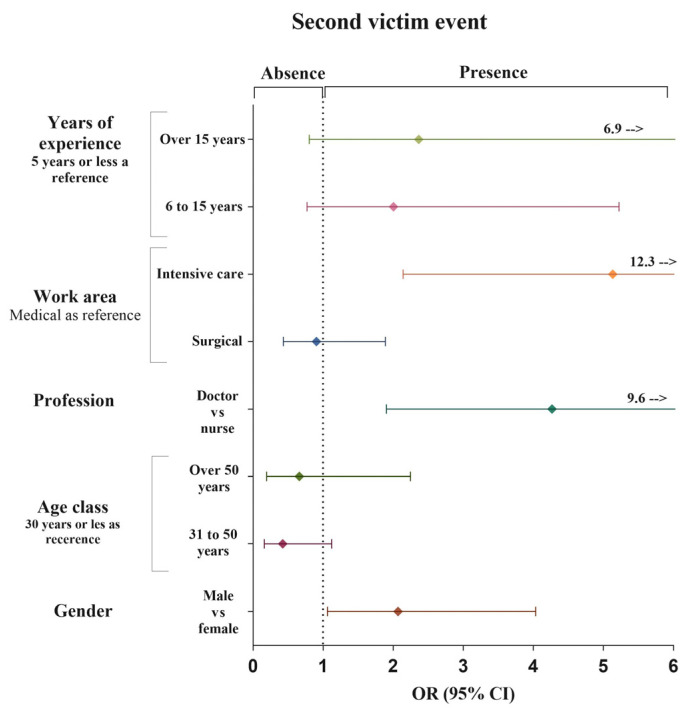
Multivariate logistic regression analysis between sociodemographic characteristics and second victim event.

The results of the multivariable binary logistic regression analysis, adjusted for age group and sex, show that being a medical doctor is positively associated with the SVE (OR = 4.267, 95% CI 1.904–9.560, *p* ≤ 0.0001). Furthermore, the event was associated with working in intensive care departments (OR = 5.133, 95% CI 2.140–12.314, *p* ≤ 0.0001) and being male (OR = 2.069, 95% CI 1.061–4.033, *p* = 0.033). However, years of professional experience and age groups were not found to be associated with the SVE (Figure 1).

**Figure 2:**
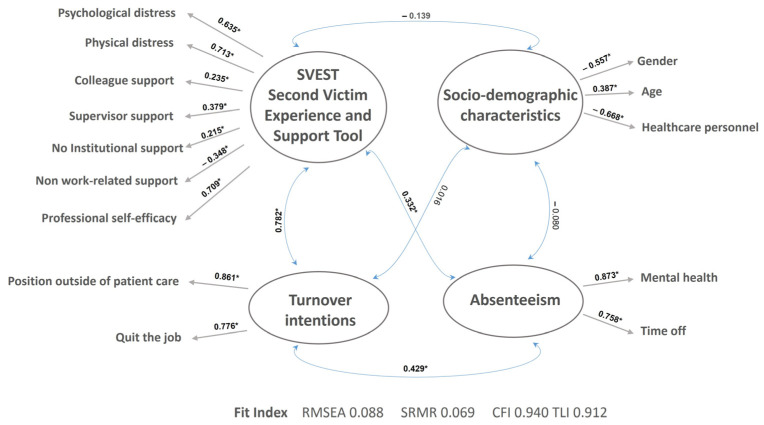
The SEM Model; RMSEA: Root Mean Square Error of Approximation, SRMR: Standardized Root Mean Square Residual; CFI: Comparative Fit Index; TLI: Tucker-Lewis Index. * Significant data (*p* ≤ 0.0001).

Figure 2 shows the standardized paths of the four components to their respective variables.

The Structural Model Fit indices indicated that the proposed model fits the data (RMSEA = 0.088, SRMR = 0.069, CFI = 0.940, TLI = 0.912). These indices demonstrate that the measurement model fits adequately [35]. All standardized paths were significant except for the path between SVEST and sociodemographic characteristics (β = −0.139, SE = 0.086, *p* = 0.106).

The SVEST component was positively associated with turnover intentions (β = 0.782, SE = 0.040, *p* ≤ 0.0001) and absenteeism (β = −0.332, SE = 0.064, *p* ≤ 0.0001). Turnover intentions were positively associated with absenteeism (β = 0.429, SE = 0.058, *p* < 0.0001) but not with sociodemographic characteristics (β = 0.348, SE = 0.137, *p* < 0.0012). Lastly, the absenteeism component was not associated with sociodemographic characteristics (β = 0.080, SE = 0.078, *p* = 0.303) (Figure 2).

## 4. Discussion

Many healthcare professionals encounter AE during their careers, which can result in SVS, lastingly impacting both their personal and professional lives [36,37].

The AE can produce effects on three different types of “victims”: the patient and their family, the HCWs involved, and the healthcare organization. This “domino effect” triggered by the event can impact other hypothetical victims, the future patients [38].

Previous studies have shown that the personal distress experienced after a medical error seems to negatively influence patient care, reducing empathy. Burnout and loss of empathy are also associated with an increased risk of future medical errors [39].

Furthermore, the fear of making a mistake often leads doctors to practice defensive medicine, prescribing unnecessary treatments and tests or avoiding high-risk patients and procedures to protect themselves from liability and the fear of being sued; this defensive approach contributes to rising healthcare costs and risks for patients [40].

Our study aimed to investigate the frequency of AE experience among HCWs at one of the largest medical centers in the Puglia Region, as well as its impact and the support systems perceived by the workers.

The prevalence of the SVE in our study is consistent with previous studies, also conducted in Italy for questionnaire validation [31,32,41], and it shows that a significant proportion of participants (41.0%) reported being involved in AE at some point in their career.

Compared to the validation studies, our research highlighted a statistically positive association between some sociodemographic characteristics, such as male gender, practicing the medical profession, and working in an intensive care unit, and the likelihood of experiencing SVS.

Although several studies did not show an association between the occurrence of the phenomenon and gender [23,30], others have suggested that female doctors may be more likely to experience negative impacts following AE compared to their male counterparts [42,43]. Other studies have instead demonstrated an association between SVS and male gender [44,45], a finding supported by previous research indicating that male doctors are at greater risk of medical malpractice than female doctors [46] and that male HWCs experience higher levels of mental stress following AE than females [45].

Furthermore, some studies have identified hospital specialties more vulnerable to AEs, such as surgery, anesthesia, pediatrics, obstetrics and gynecology [47], and intensive care, where seriously ill patients undergo invasive procedures [48].

HCWs involved in AE may experience both psychological and physical impacts that can impair their job performance and patient safety [17,49].

The literature highlights a range of symptoms commonly observed among HCWs involved in AE, including insecurity, lower professional self-efficacy, guilt, and fear of damaging their reputation [3,49]. In extreme cases, these impacts can lead to the desire to abandon the profession [50] or even thoughts of suicide [51]. In our sample, most participants reported feelings of embarrassment, unhappiness, and insecurity about future events. These psychological manifestations are closely linked to physical symptoms, with many respondents describing feelings of exhaustion, as well as experiencing sleep disturbances and appetite changes following the AE.

Numerous studies have listed depression as one of the effects of SVS [3,37]. A recent review also showed an association between a physician’s depressive symptoms and an increased risk of medical errors [52]. Brunsberg et al. claimed that an HCW suffering from depression is three times more likely to make harmful errors compared to their colleagues [53].

The effects of SVS are influenced by personal, interpersonal, and environmental factors, which can either amplify or mitigate their impact [23]. In our study, nearly one in three participants reported feeling inadequate and doubting their professionalism after an AE, with 40.0% expressing a desire to change jobs.

Low professional self-efficacy and lack of support were identified as key factors linked to turnover and absenteeism [54,55].

Our findings revealed a correlation between work-related outcomes and all dimensions of SVEST, while no significant association was found with sociodemographic variables, in accordance with the results of recent studies [56,57]. This result suggests that the intention to leave one’s job or take a leave of absence is primarily driven by the severity of the emotional distress and the personal impact experienced by the second victim.

Consistent with previous studies, nearly all participants identified opportunities to talk and share difficult experiences with others as key coping mechanisms [58,59].

Regarding the support received, in the majority of cases, the operators preferred to discuss the AE with colleagues. Talking to a colleague about what happened helped alleviate negative emotions, calm feelings of guilt or insecurity, reduce the sense of isolation, and reassure the fear of judgment, especially because it is very likely that we have had similar experiences [4]. The second most sought and received form of support was non-work-related. Although friends and family play an important role in these situations due to their emotional closeness, their ability to provide adequate support may be limited by an incomplete understanding of the medical and hospital dynamics [24].

Paradoxically, while most participants expressed a desire for support programs within their organization, the literature data describe multiple obstacles that prevent operators from seeking institutional support. These obstacles include fears about confidentiality, potential impact on their career, lack of time, and the stigma surrounding mental health care [24].

Frequently, they chose peer-to-peer support that occurs naturally between colleagues rather than relying on a formal program. However, for these supportive relationships to be effective, the peer providing support must have the necessary skills to offer adequate assistance [13].

To date, few institutions have implemented peer support programs to address the post-AE experience and the associated emotional labor [60,61]. However, providing adequate support for second victims is recognized as a critical safety standard by major national and international organizations; the WHO includes it in strategic plans for patient safety [62].

It is well known that hospitals with an SV management approach tend to foster a stronger culture of quality and safety, which, over time, reduces adverse events [63].

HCWs who engage in these programs are more likely to make constructive changes in their behavior [64,65], enhancing the organization’s safety culture [66] and promoting professional growth and recovery from SVS, what Scott et al. define as the sixth phase of “thriving” [3]. Such programs also offer economic benefits to healthcare institutions by reducing system-related costs, absenteeism, and turnover rates, leading to improved quality patient care [67].

Raising awareness of the “second victim” phenomenon, its causes, and its effects is crucial to breaking the culture of silence in healthcare and the isolation of HCWs, which are the main barriers to recovery strategies [58,68].

SVS can result from various clinical events, not just actual or missed medical errors [69]. Unexpected or adverse patient outcomes may occur due to disease progression or complications beyond the healthcare provider’s control [70] or from violent encounters with patients, an increasingly widespread phenomenon [71].

Understaffing, particularly evident during the COVID-19 pandemic, is a significant risk factor for SVS [72,73,74]. In addition to work-related stress, accusations or complaints from patients, colleagues, or supervisors, negligence lawsuits are added to the list of events capable of triggering SVS [70,75], so that the so-called “clinical-judicial syndrome” is considered one of the most emotionally damaging experiences for a physician [36].

A key factor influencing the severity of SVS is the presence of a punitive safety culture within healthcare settings [49]. Addressing this issue requires a cultural shift toward a supportive approach that prioritizes the psychosocial well-being of the second victim [63]. It is essential to recognize systemic factors and learn from these incidents to foster a just culture [76].

Further research is needed to identify high-risk events, effective strategies to manage the aftermath of an incident, surveillance systems, and timely and proactive support for HCWs exposed to the risk of SVS.

Our study is not without limitations. Although we achieved a good response rate, the majority of participants were nurses, as is commonly observed in most research [36]; this reflects their larger representation in hospital staff [77] and the tendency of doctors to respond less to surveys [78].

The voluntary nature of participation and the use of a convenience sample may have introduced selection bias, and HCWs who chose to participate were probably more sensitive to the topic.

Although the survey was anonymous, some respondents may not have disclosed their involvement in an AE because of prejudice and social stigma. Yaow et al. noted that only a small percentage of HCWs (21.4%) reported being involved in an AE due to fears of litigation and retaliation, as well as the absence of a clear definition of reportable AE [23].

In our study, we utilized a validated questionnaire; however, we believe that it requires some further refinement. For instance, it would be beneficial to include questions regarding knowledge of the second victim phenomenon and to provide a clearer definition for terms such as “adverse event” and “near miss”. The low reporting of “near misses” (1 in 250) suggests confusion around these terms. Additionally, the questionnaire did not allow us to assess the severity of AEs or track the evolution of SVS over time.

These factors could have influenced the results concerning the prevalence rate, even though our findings are similar to those of other studies from central and northern Italy [29,30] and most international studies on the topic of the second victim [26,27,28].

Overall, our findings confirmed that distress experienced by HCWs due to SVS is debilitating on an individual level and can impact their performance, with implications for patient care, healthcare organizations, and the healthcare system as a whole.

We have limited knowledge about how professionals deal with AE. Currently, there is no formal support system in place at our center, nor is there a requirement within the Italian National Health Service (NHS). This information is essential to increase awareness among institutions about the issue and encourage them to implement specific action.

## 5. Conclusions

AE poses a significant public health concern. Sudden complications and unintentional errors are common in clinical practice due to natural human fallibility. HCWs face complex situations daily and may encounter unexpected outcomes, which can compromise not only the health of patients but also that of HCWs themselves. SVS is a serious issue that involves the entire healthcare system. Although some institutions in Western countries have developed formal support programs to help HCWs affected by SVS, there is a strong need to increase awareness of the phenomenon, provide appropriate institutional responses, and develop competences for peer-to-peer support.

A work environment that promotes psychological support and reduces the stigma associated with errors is an environment in which professionals are more attentive and focused, reducing the risk of errors and improving patient safety.

## Figures and Tables

**Table 1:** Sociodemographic characteristics of the respondents.

				Involvement in an AE				
		Total (n = 246)		No (n = 145)		Yes (n = 101)		
		n°	%	n°	%	n°	%	*p*-Value
Gender	Male	70	28.9	31	21.7	39	39.4	0.003
	Female	172	71.1	112	78.3	60	60.6	
Age groups (years)	≤30	51	21.0	30	21.0	21	21.0	0.395
	from 31 to 50	125	51.4	78	54.5	47	47.0	
	>50	67	27.6	35	24.5	32	32.0	
Profession	Doctor	46	19.3	16	11.6	30	30.0	0.0001
	Nurse	192	80.7	122	88.4	70	70.0	
Work area	Medical	128	54.7	78	56.9	50	51.5	0.003
	Surgical	66	28.2	45	32.8	21	21.6	
	Intensive care	40	17.1	14	10.2	26	26.8	
Experience (years)	≤5	59	24.4	36	24.4	23	23.0	0.740
	From 6 to 15	74	30.6	45	31.7	29	29.0	
	>15	109	45.0	61	43.0	48	48.0	

**Table 2:** Agreement, Means, SDs, and n. Items of the I-SVEST Dimensions and Outcome Variables.

		Agreement (%)	Mean	SD	Items
Dimensions	1. Psychological Distress	23.5	3.3	0.78	4
	2. Physical Distress	9.9	2.8	0.78	4
	3. Colleague Support	1.0	2.6	0.51	4
	4. Supervisor Support	9.3	2.9	0.67	4
	5. No-Institutional Support	23.8	3.3	0.68	3
	6. Non-Work-Related Support	9.9	2.4	0.82	2
	7. Professional Self-Efficacy	8.0	2.8	0.79	2
Outcome	8. Turnover Intentions	19.8	2.7	1.09	2
	9. Absenteeism	8.9	2.3	0.90	2

**Table 3:** I-SVEST items and outcome variables.

Question	% Agreement
1. I have experienced embarrassment from these instances.	54.0
2. My involvement in these types of instances has made me fearful of future occurrences.	48.5
3. My experiences have made me feel miserable	55.5
4. I feel deep remorse/guilt for my past involvement in these types of events.	41.4
5. The mental weight of my experience is exhausting.	51.5
6. My experience with these occurrences can make it difficult to sleep regularly	33.7
7. The stress from these situations has made me feel queasy or nauseous.	23.8
8. Thinking about these situations can make it difficult to have an appetite.	14.9
9. I appreciate my coworkers’ attempts to console me, but their efforts can come at the wrong time.	44.6
10 Discussing what happened with my colleagues provides me with a sense of relief.	78.2
11. My colleagues can be indifferent to the impact these situations have had on me.	26.7
12. My colleagues help me feel that I am still a good healthcare provider despite any mistakes I have made.	75.0
13. I feel that my supervisor treats me appropriately after these occasions.	42.6
14. My supervisor’s responses are fair.	30.3
15. My supervisor blames individuals.	36.0
16. I feel that my supervisor evaluates these situations in a manner that considers the complexity of patient care practices.	44.0
17. My organization understands that those involved may need help to process and resolve any effects they may have on care providers.	46.5
18. My organization offers a variety of resources to help me get over the effects of involvement with these instances.	22.8
19. The concept of concern for the well-being of those involved in these situations is not strong at my organization.	54.5
20. I look to close friends and family for emotional support after one of these situations happens.	64.4
21. The love from my closest friends and family helps me get over these occurrences.	70.3
22. Following my involvement I experienced feelings of inadequacy regarding my patient care abilities.	36.6
23. My experience makes me wonder if I’m not really a good healthcare provider.	29.7
24. After my experience, I became afraid to attempt difficult or high-risk procedures.	31.7
25. These situations don’t make me question my professional abilities.	54.0
26. My experience with these events has led to a desire to take a position outside of patient care	2.8
27. Sometimes the stress from being involved with these situations makes me want to quit my job	40.6
28. My experience with an adverse patient event or medical error has resulted in me taking a mental health day	11.9
29. I have taken time off after one of these instances occurs.	24.8

**Table 4:** I-SVEST Support Option.

Question	% Agreement
1. The ability to immediately take time away from my unit for a little while.	32.7
2. A specified peaceful location that is available to recover and recompose after one of these types of events	51.0
3. A respected peer to discuss the details of what happened.	69.0
4. An employee assistance program that can provide free counseling to employees outside of work.	78.2
5. A discussion with my manager or supervisor about the incident.	68.0
6. A confidential way to get in touch with someone 24 h a day to discuss how my experience may be affecting me	62.4
7. The opportunity to schedule a time with a counselor at my hospital to discuss the event	63.4

## Data Availability

The original contributions presented in the study are included in the article, further inquiries can be directed to the corresponding author.
